# Uptake and determinants for HIV postpartum re-testing among mothers with prenatal negative status in Njombe region, Tanzania

**DOI:** 10.1186/s12879-019-4062-8

**Published:** 2019-05-09

**Authors:** Saumu Iddy Nungu, Janneth Maridadi Mghamba, Susan Fred Rumisha, Innocent Antony Semali

**Affiliations:** 1 Tanzania Field Epidemiology and Laboratory Training Program, P. O. Box 9083 Dar es Salaam, Tanzania; 2 Ministry of Health, Community Development, Gender, Elderly, and Children, P. O. Box 743 Dodoma, Tanzania; 3 Muhimbili University of Health and Allied Sciences, P. O. Box 65001 Dar es Salaam, Tanzania; 4 National Institute for Medical Research, 3 Barack Obama Drive, P. O. Box 9653 Dar es Salaam, Tanzania

**Keywords:** Retesting, Uptake, Postpartum, Prenatal, HIV, Tanzania

## Abstract

**Background:**

Uptake of Human Immunodeficiency Virus (HIV) re-testing among postnatal mothers who had previously tested HIV-negative is crucial for the detection of recent seroconverters who are likely to have high plasma viral loads and an increased risk of mother-to-child HIV transmission. Tanzania set a target of 90% re-testing of pregnant mothers who had tested negative during the first test. However, there is no statistics on the implementation, coverage and the factors determining re-testing among pregnant women in Tanzania. This study determined the proportion of newly-delivered, previously HIV-negative mothers who returned for HIV re-testing, and assessed the determinants of re-testing in Njombe Region in Tanzania.

**Methods:**

A cross-sectional study was conducted in four health facilities in Njombe and Wanging’ombe districts during December 2015–June 2016. All newly-delivered mothers (≤7 days from delivery) presenting at health facilities and who had previously tested HIV-negative during pregnancy were included. A structured questionnaire was used to collect data on the determinants for re-testing. Records on the previous HIV testing was verified using antenatal clinic card. A multiple logistic regression model was used to calculate the adjusted odds ratio (AOR) with their 95% confidence intervals (CI) to quantify the association.

**Results:**

Of 668 mothers (median age = 25 years) enrolled, 203 (30.4%) were re-tested for their HIV status. Among these, 27 (13.3%) tested positive. Significant predictors for HIV re-testing were socio-demographic factors including having at least a secondary education [AOR = 1.9, 95% CI: 1.25–3.02] and being employed [AOR = 2.1, 95% CI: 1.06–4.34]; personal and behavioural factors, reporting symptoms of sexually transmitted infections [AOR = 4.9, 95% CI: 2.15–6.14] and use of condoms during intercourse [AOR = 1.7, 95% CI: 1.13–2.71]. Significant health system factors were having ≥4 ANC visits [AOR = 1.8, 95% CI: 1.21–2.69] and perceiving good quality of HIV counselling and testing service at the first ANC visit [AOR = 2.14, 95% CI: 1.53–3.04].

**Conclusion:**

Uptake of the HIV re-testing was lower than the national target. Education level, employment status, having ≥4 ANC visits, reporting sexually-transmitted infections, condom use, and good perception of HIV tests were significant factors increased uptake for re-testing. Identified factors should be incorporated in the Prevention of the Mother-to-Child Transmission (PMTCT) programme strategies to prevent HIV infection in new-borns.

## Background

The Human Immune-deficiency Virus (HIV)/Acquired Immune Deficiency Syndrome (AIDS) constitutes a serious public health problem. Globally, 36.9 million people live with HIV, out of whom 1.8 million people are newly-infected cases [1]. Of these about 70% are adults and children who live in Sub-Saharan Africa (SSA). The SSA region has about 25.8 million people living with HIV which can cause AIDS [2]. In 2017, women accounted for almost 59% (578,200 million) of the adults living with HIV in SSA. During the same time, 1.8 million children aged 0–14 years were living with HIV with 180,000 being newly-infected children, hence indicating high risk of HIV exposure to unborn babies and infants [1].

Global response to the HIV pandemic include the adoption of Sustainable Development Goal 3 targets and strategies for better health for mothers and their children [3]. The goal aims to ensure healthy lives and promote the wellbeing for all at all ages through the prevention of deaths among new-borns and ending the AIDS epidemic by 2030 [4]. The Prevention of the Mother-to-Child Transmission (PMTCT) of HIV remains as one of the major preventive strategies in many SSA countries. Efforts to stop the rising infection rates of HIV include integrating PMTCT services with the routine maternal, neonatal and child health (MNCH) services [13]. However, PMTCT roll-out has faced a number of challenges including social stigma, fears to lose friends, discrimination and other factors which discourage the uptake of HIV testing and re-testing [28].

Tanzania is one of the 22 countries with the highest number of pregnant mothers living with HIV [5]. Among the country’s 50 million people, 1.5 million are living with HIV. Of these, about 810,000 are women and 120,000 are children under the age of 14 years [6]. Among the children with HIV infection, about 90% acquired the virus through vertical transmission [7]. The mother-to-child transmission of HIV remains high despite the recent country’s achievements in reducing HIV prevalence [7]. In 2010, the government of Tanzania adopted the World Health Organisation (WHO) PMTCT Guidelines Option B^+^ and facilitated the adoption of strategies aimed to overcome barriers to effective PMTCT programme implementation, including expanding access to treatment for pregnant mothers [7]. Option B+ recommends for the administration of anti-retroviral treatment to pregnant or postpartum mothers for life. This has resulted in early HIV diagnosis for a larger number of the mothers and, therefore, facilitating the utilisation of the interventions to halt the mother-to-child HIV transmission [8].

The PMTCT process involves HIV testing and counselling at the onset and subsequent administration of antiretroviral therapy (ART) to HIV-positive cases. However, those who are HIV-negative are counselled to return for a HIV re-testing three months after the first test, if missed, be re-tested during labour or immediately after delivery [7]. Moreover, in settings where the HIV epidemic is generalised testing is recommended during the third trimester or at delivery [9, 18–24]. The success of PMTCT depends inter alia on the readiness of the targeted women to undergo testing for initial HIV testing and thereafter retesting if initially the HIV sero-status was negative. However, experience from a number of countries shows that HIV re-testing for pregnant mothers is rarely implemented [9].

To achieve the goal of zero mother-to-child transmission of HIV, immediate strategies should focus on the promotion of sustainable HIV re-testing within the PMTCT programme. That notwithstanding, valid information on current uptake and facilitating factors need to be known. In Tanzania, there is dearth of information relating to the extent of HIV re-testing among pregnant mothers or those who have just delivered, previously found to be HIV-negative and were eligible for re-testing. Moreover, factors influencing previously HIV-negative delivered mothers to go for HIV re-testing are not known with certainty. Thus this study was carried out to determine the re-testing uptake and the determinants of HIV re-testing in a rural region of Njombe in Tanzania. This knowledge is crucial in informing the current PMTCT strategies and policies.

## Methods

### Study design and setting

This health facility-based cross-sectional study was carried out in Njombe Region, Tanzania. The region is located in the south-western highlands of Tanzania. Njombe region comprises four districts of Njombe, Ludewa, Makete, and Wanging’ombe with a total area of 21,347km^2^. The region is served by 221 health facilities of which four are hospitals, 22 health centres and 193 dispensaries. Njombe is one of the regions with high HIV prevalence (15.4%) among adults in Tanzania [12].

Tanzania adopted the Focused Antenatal Care (FANC) model in all its health facilities, which recommends that a pregnant woman makes fewer visits starting at 16 weeks, a second visit between 20 and 24 weeks, a third visit between 28 and 32 weeks, and a fourth visit at 36 weeks. All health centres and hospitals with comprehensive PMTCT programmes have been integrated to follow this model when providing antenatal care for expectant mothers. Those who test HIV-negative are counselled and advised to repeat the test during the third trimester or at labour and delivery [7].

### Study population and criteria for participation

**Fig. 1:**
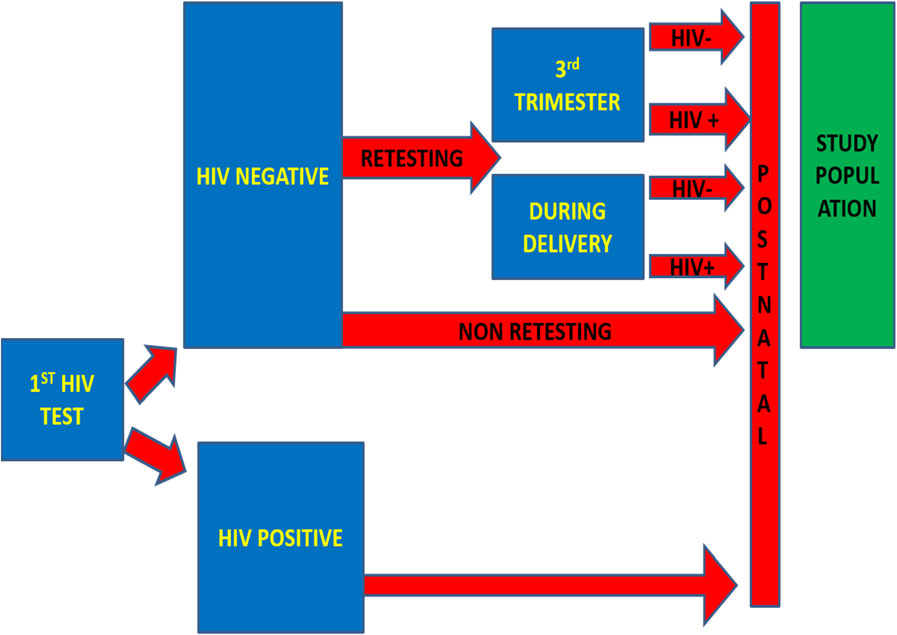
Diagrammatic presentation of the study population

The study population included all the pregnant mothers who had just delivered at the selected health facilities from 14th December 2015 to 12th February 2016. The inclusion criteria were those women who had HIV-negative during their first test with at least three months from the first HIV test. All the mothers who were severely ill at postnatal, those with mental problems and the deaf were excluded from the study. (Fig. 1).

### Sample size determination

Sample size was determined based on the formula for cross-sectional studies [38]. It was assumed that the proportion (*p*) of HIV-negative mothers at first antenatal HIV test who accepted HIV re-testing before delivery, during delivery or immediately after delivery was 50% with 4% marginal of error (*E*). Precision was set at 0.05 of which *Z* = 1.96, the statistic corresponding to 95% level of confidence. We assumed a conservative re-testing rate of 50% for the negative tested women as we could not establish a relevant reference with the actual rate for this population. The calculated sample size was then adjusted for 10% non-response . Subsequently, a minimum of 600 sample size of women was obtained using the formula.

### Sampling technique

Multistage sampling technique was conducted. During the first stage, all the districts in Njombe region were listed before two were randomly selected using the lottery methodology. The selected districts were Wanging’ombe and Njombe. During the second stage, one hospital was selected by default (as only one existed) and one health centre was selected using the lottery method from each selected district. Kibena hospital and Njombe health centre were chosen from Njombe District whereas Ilembula Hospital and Wanging’ombe Health Centre were chosen from Wanging’ombe District. At the selected facilities, all the mothers who had delivered during the study period and met the set criteria were recruited sequentially as they arrived without skipping until the required sample size had been reached.

### Pre-testing of the tool

The questionnaire was first developed in English and then translated into Kiswahili, it was then pretested at Kipengele Health Centre (not included in the study sample) before the commencement of the actual data collection. The research tool was revised to ensure that its questions capture reliable information and to improve clarity.

### Recruitment and training of research assistants

Four research assistants, who were enrolled nurses working in Njombe public health facilities were recruited and trained for three days on the study objectives, study methodology, interview techniques, research ethics and data collection tools. The enrolled nurses were useful in because reviewing information recorded on the ANC card, hence making them ideal research assistants. All the research assistants were fluent in Kiswahili, the language deployed in data collection as it was accessible and understandable to all the mothers who took part in the study.

### Data collection and variables

Immediately after delivery and before being discharged, the ANC card of each postpartum mother was retrieved and those who met the inclusion criteria were included in the study. The information extracted from the card was the respective mother’s HIV re-testing status, time for re-testing and HIV results at re-testing. The mothers were asked about their HIV re-testing and their response were verified against the records on their respective ANC card. If there were some inconsistency in the information, the one on the ANC card was taken as a reliable answer. The re-testing status was the main outcome of the study for which a binary variable (0 if no re-testing and 1 if it was done) was created. All the information was recorded on a standard form and later linked with the main questionnaire. The main questionnaire collected data on the following variables: socio-demographic characteristics such as the age of the mother, occupation, marital status, education level, partner’s education level; mother’s obstetric factors including gestation age at booking, number of ANC visits, parity; attitude, practice and perceived risk of HIV including condom use, reported symptoms of sexually-transmitted infections (STIs); perceived quality of counselling during first HIV test; and perceived severity of HIV. Both perceived quality of counselling and perceived satisfaction with ANC services were measured using a set of questions developed using a 5-point Likert scale (*Strongly agree, Agree, Uncertain, and Disagree and Strongly disagree*). There were 21 questions on quality and 5 on satisfaction.

For each of the question, a new binary variable was created which collapsed those responses with *Strongly agree* and *Agree* (as they were treated as satisfied) against the rest (treated as not satisfied).

The reliability test for the set of questions for each domain was done using the Cronbach’s alpha internal consistency test. Value greater than 0.7 for the Cronbach’s alpha was regarded as acceptable. Acceptable questions were subjected to Principal Component Analysis (PCA) with varimax rotation to create components and respective factor loading. In each domain (perceived quality of counselling and perceived satisfaction), the first component which also accounted for most of the variance was adopted as the variable in that respective domain. The new variables were converted into dichotomous variables with two levels.

### Data management and analysis

Data was double entered into the Epi info 3.1.5 template, with data cleaning done by visual inspection and logic check. Analysis was done using Epi info 3.1.5 (CDC, Atlanta, GA, USA) and STATA version 12 (Statcorp, College Station, TX, USA). Univariate analysis was done by performing frequency and cross-tabulations to indicate the distribution of socio-demographic characteristics of the respondents and determine the proportion of the mothers who had HIV re-testing. Bivariable analysis was carried out to determine the association of the outcome variables (uptake HIV re-testing) with the predictor variables. Then the chi-square statistic was done to establish the significance of association. Finally, multiple logistic regression was performed to identify the significant predictors contributing to the uptake of HIV re-testing while controlling for other factors. All the variable with *p* < 0.2 in bivariable analysis were added to the multiple logistic regression model. In all the analyses, the statistical significance was considered when *p*-value was < 0.05.

## Results

### Socio-demographic and obstetric characteristics of respondents

The study involved 668 women who had given birth and qualified for inclusion. Their median age was 25 years (range 15–45 years) (Table 1). The majority were married (82.2%), unemployed (91.2%) and had low educational level (64.2%). About two-thirds (66.3%) reported a parity of less than 3 whereas the majority (86.6%) booked their first ANC visit before the end of second trimester. However, most (56.3%) had less than four ANC visits before delivery (Table 1).

**Table 1:** Socio-demographic and obstetric characteristics of respondents

Variable	Number(*N* = 668)	Percentage (%)
Maternal age (years)		
25+	345	51.6
< 25	323	48.4
Educational status		
High Education	239	35.8
Low education	429	64.2
Employment status		
Employed	59	8.8
Unemployed	608	91.2
Marital status		
Married/ Cohabiting	549	82.2
Single/ Divorced/ Separated	119	17.8
Partner’s education level		
High education	264	39.5
Low education	404	60.5
Parity		
≤3	443	66.3
>3	225	33.7
Gestation at booking		
1st and 2nd trimester	578	86.5
3rd trimester	134	13.5
Number of ANC visits		
≥4	292	43.7
< 4	376	56.3

### Uptake of HIV re-testing

**Fig. 2:**
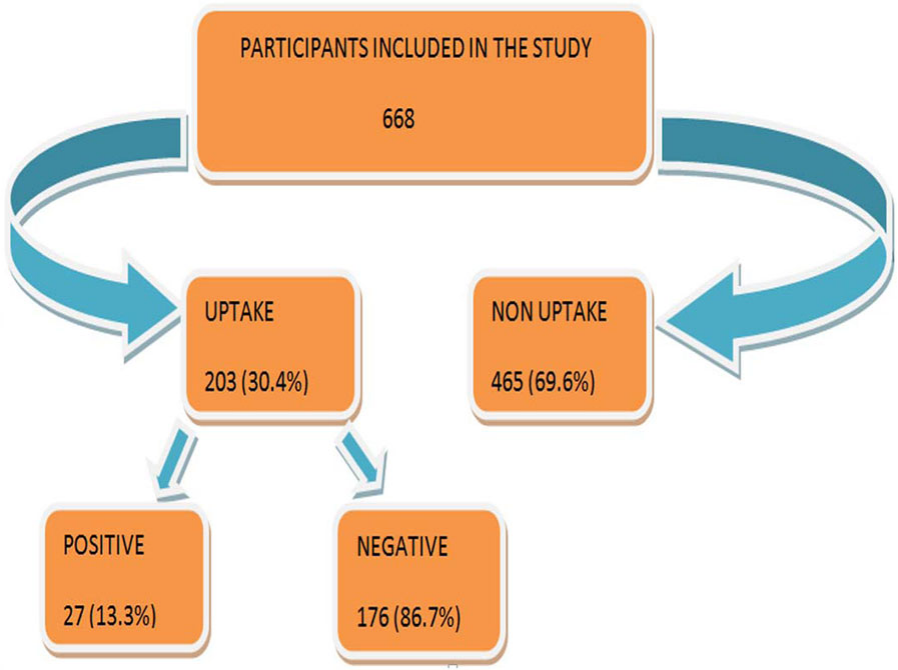
Uptake of HIV re-testing

Less than a third (30.4%) of the previously HIV-negative mothers had re-tested for HIV of whom 27 (13.3%) were found to be HIV-positive (Fig. 2). Furthermore, those who were married (15.5%) compared to those who were not married (2.9%) had a higher likelihood of being HIV-positive. The difference was statistically significant (χ^2^ = 2.98, *p* = 0.03). On the other hand, other social and demographic variables were not statistically significant (Table 2).

**Table 2:** Distribution of HIV status by socio-demographic characteristics

Variable	HIV status (%)		*P* value
	Positive	Negative	
Maternal age			
25+	10(11.0)	81(98.0)	0.38
<25	17(15.2)	95(84.8)	
Educational status			
High Education	15(15.5)	82(84.5)	0.39
Low education	12(11.3)	94(88.7)	
Employment status			
Employed	3(9.4)	29(90.6)	0.35
Unemployed	24(14.0)	147(86.0)	
Marital status			
Single/Divorced/Separated	1(2.9)	34(97.1)	
Married/Cohabited	26(15.5)	142(84.5)	0.03
Partner’s education level			
High education	15(16.9)	74(83.1)	0.19
Low education	12(10.5)	102(89.5)	

### Perception of the integrated PMTCT services

Half (49.6%) of the respondents were satisfied with ANC services; two-thirds (66.3%; *n* = 443) had a perception that the quality of HIV testing at the ANC was good. On the other hand, slightly less than half (45.2%; *n* = 302) of the mothers reported perceiving as being at risk of HIV infection (Table 3). The majority 593 (88.8%) of the respondents perceived HIV as severe disease; less than a quarter (14.5%; *n* = 97) of the mothers were reported to have symptoms of sexually-transmitted infections during pregnancy; and 25.4% (*n* = 170) of the respondents were using condom during sexual intercourse.

**Table 3:** Participants’ clinical information and perception of the service provided

Variable	Frequency (*N* = 668)	Percentage (%)
Perceived satisfaction with ANC services		
Satisfied	331	49.6
Not satisfied	337	50.4
Perceived quality of HIV testing		
Good perception	443	66.3
Bad Perception	225	33.7
Perceived risk of HIV		
Yes	302	45.2
No	366	54.8
Perceived severity of HIV		
Yes	593	88.8
No	75	11.2
Reported symptoms of STI		
Yes	97	14.5
No	571	85.5
Condom Use		
Yes	170	25.4
No	498	74.6

### Association of uptake HIV re-testing with socio-demographic and obstetric characteristics

A larger proportion (40.2%) of the mothers with higher education went for HIV re-testing than those with low education (24.9%) (χ^2^ = 16.82, *p* < 0.001) (Table 4). Among those who were employed, a significantly higher proportion (52.5%) went for HIV re-testing than those (28.2%) who were unemployed (χ^2^ = 15.01, *p* < 0.001). Other factors that showed significant association with re-uptake of HIV test were parity (χ^2^ = 4.85, *p* = 0.028) and number of ANC visits (χ^2^ = 10.67, *p*-value < 0.001).

**Table 4:** Bivariable analysis of the association uptake HIV re-testing with socio-demographic and obstetric characteristics

Variable	Uptake HIV re-testing n (%)		Chi-square	P value
	Yes	No		
Maternal age				
25+	91 (28.2)	232 (71.8)	1.45	0.228
<25	112 (32.5)	233 (67.5)		
Educational status				
High Education	96(40.2)	143(59.8)	16.82	< 0.001
Low education	107(24.9)	322(75.1)		
Employment status				
Employed	31(52.5)	28(47.5)	15.01	< 0.001
Unemployed	172(28.2)	437(71.8)		
Marital status				
Single/Divorced/Separated	35 (29.4)	84 (70.6)		
Married/Cohabited	168 (30.6)	381 (69.4)	0.07	0.798
Partner’s education level				
High education	88 (33.3)	176 (66.7)		
Low education	115 (28.5)	289 (71.5)	1.79	0.18
Parity				
≤3	147 (33.2)	296 (66.8)		
>3	56 (24.9)	169 (75.1)	4.85	0.028

### Association of uptake HIV re-testing with clinical/ individual factors

Table 5 presents the results on the association of HIV re-uptake with selected aspects of the quality of services on offer. Those who perceived the quality of HIV testing services to be good had a significantly higher proportion (35.7%) taking the HIV re-test than those (20.0%) who perceived otherwise (χ^2^ = 17.3, *p* < 0.001). Those who perceived to be at high risk of HIV infection had a significantly higher proportion (34.8%) of retesting for HIV than those (26.8%) who perceived to be at lower risk (χ^2^ = 4.99, *p* = 0.025). Other factors which had significant associations were reporting past STI symptoms (χ^2^ = 49.69, *p* < 0.001) and condom use (χ^2^ = 6.64, *p* = 0.009) (Table 5).

**Table 5:** Bivariable analysis of the association of uptake HIV re-testing with clinical/ individual factors

Variables	Uptake HIV re-testing n (%)		*P* value
	Yes	No	
Perceived Satisfaction of ANC services			
Satisfied	104 (31.4)	227 (68.6)	
Not satisfied	99 (29.4)	238 (70.6)	0.57
Perceived quality of HIV testing			
Good perception	158(35.7)	285(64.3)	
Bad perception	45(20.0)	180(80.0)	< 0.001
Perceived risk of HIV			
Yes	105 (34.8)	197 (65.2)	0.025
No	98 (26.8)	268 (73.2)	
Perceived severity of HIV			
Yes	181 (30.5)	412 (69.5)	0.83
No	22 (29.3)	53 (70.7)	
Reported symptoms of STI			
Yes	59 (60.8)	38 (39.2)	
No	144 (25.2)	427 (74.8)	< 0.001
Condom Use			
Yes	65 (38.2)	105 (61.8)	0.009
No	138 (27.7)	360 (72.3)	

### Factors associated with HIV re-test uptake

Table 6 presents independent variables which were significantly associated with a pregnant woman returning for HIV re-testing. The multivariable analysis results revealed that mothers with at least secondary education were two times more likely to go for HIV re-testing than those with lower education (AOR = 1.9, 95% CI 1.25–3.02). Employed mothers were two times more likely to go for HIV re-testing than the unemployed ones even after adjusting for potential confounders (AOR = 2.1, 95% CI 1.06–4.43). Similarly, after adjusting for confounding variables, mothers with four or more ANC visits were two times more likely to go for HIV re-testing than those with less than four ANC visits (AOR = 1.8, 95% CI1.21–2.69). Overall, the uptake of HIV re-testing were five times likely among mothers who had reported symptoms of STIs than those who had not (AOR = 4.9, 95% CI 2.15–6.14). Mothers who had ever used condom were two times more likely to go for HIV re-testing than those who were not using condoms (AOR = 1.7, 95%CI1.13–2.71). Moreover, the uptake of HIV re-testing was two times more likely to occur among mothers who perceived HIV services at the ANC to be of good quality than those who perceived otherwise (AOR = 2.14(1.53–3.04) 95% CI).

**Table 6:** Factors associated with HIV re-testing uptake

Variable	Crude OR (95%Cl)		AOR (95%Cl)	
Educational status (Reference = Low education)				
High Education	2	(1.42–2.81)	1.9	(1.25–3.02)
Employment status (Reference = Unemployed)				
Employed	2.8	(1.57–4.75)	2.1	(1.06–4.34)
Parity (Reference = 3+)				
< 3	1.5	(1.02–2.21)	1.2	(0.81–1.83)
Number of ANC visit (Reference = < 4)				
> 4	1.7	(1.20–2.42)	1.8	(1.21–2.69)
Reported Symptoms of STI (Reference = No)				
Yes	4.6	(2.86–7.20)	4.9	(2.15–6.14)
Condom Use (Reference = No)				
Yes	1.6	(1.10–2.32)	1.7	(1.13–2.71)
Perceived quality of HIV testing (Reference = No)				
Yes	2.22	(1.48–3.20)	2.1	(1.53–3.04)
Perceived severity of HIV (Reference = No)				
Yes	1.5	(1.11–2.03)	1.0	(0.74–1.52)

## **Discussion**

This study assessed returning rate for re-testing, among pregnant women who tested HIV-negative at first ANC test and had at least three months to delivery. Significant determinants for uptake of HIV re-testing were mother’s education level, employment status, having more than four ANC visits, reported having STIs, using condoms and those who perceived quality of the initial HIV test to be of good quality.

In this study, only a third of pregnant women who tested negative at first test re-tested. The uptake rate of was below the recommended PMTCT level of above 90% [7] and in spite of the mothers being in contact with health facilities during antenatal care and delivery. The proportion of returning for HIV re-testing observed in this study is similar to what was reported in a study conducted in Zambia which found that only 1-out-of-4 women was re-tested [33]. Though in anticipation HIV re-uptake among mothers who had delivered could be higher than that of the general population due to their frequent visits to healthcare facilities that was not the case in this study. Population and community-based studies with comparable findings on re-testing rates have been reported in South Africa [15], Croatia [16] and Vietnam [17]. This low utilisation could be explained by client and health system factors. Client factors could be trust in the initial test results, spousal influence and perceptions of stigma as well as discrimination. Self-confidence and risky behaviour were also explained by condom use hence could be related to why most of those who tested negative did not see the need for re-testing. In this study, condom users were more likely to opt for HIV re-test than those who were not using condoms during sexual intercourse. This has also been observed in other studies which reported a higher uptake of HIV re-test services among participants who had reported condom use [16, 35] or had involved in less risky sexual activities [29]. This situation could be attributable to the high perceived risk among condom non-users which, in turn, made them dread the test and, hence, avoid returning for the re-testing. Personal health status also influences the re-testing decision as it was observed that individuals reporting symptoms of STIs had higher uptake rate. Similar results have been reported in Thailand [34] and Croatia [16]. HIV retesting in our study was lower than a similar study conducted in Nairobi Kenya, which revealed the feasibility and acceptance of HIV re-testing during labour and postpartum [22]. Health system factors that have been reported to affect re-testing coverage include availability of supplies, limited structural quality, negative perception of providers and inability to impart adequate and effective requisite knowledge among clients during counselling. Consequently, the promotion of HIV re-testing among HIV-negative mothers should target both client and health system factors. However, most mothers reported to be satisfied with the service and, though this was not the focus for this study, no complaints on unavailability of testing kits were reported.

The finding that mothers with higher education levels were about twice more likely to return for HIV re-testing than those without or with lower education was generally not surprising. It has been observed that people with better education generally have better access to information and knowledge which boosts their utilisation of health services [25]. In addition, educated pregnant mothers would have a better understanding and internalisation of HIV counselling messages acquired during their first test than those without or with low education. As a result, these educated mothers were able to make individual decision of complying and, consequently, going back to the health facility for the HIV re-testing. These results are consistent with those of other studies elsewhere which showed that those with low education level were more likely to refuse HIV test [27] whereas those with intermediate and high education levels presented high levels of acceptance [26].

Pregnant women who had made more than four visits had higher likelihood for re-testing for HIV. Implicitly, more than four ANC visits would also imply early ANC booking, thus providing adequate time to undergo the first HIV test and increase the likelihood of re-testing three months later before delivery. Late bookings have also been observed to result in fewer ANC visits and high possibility to deliver before three months necessary for re-testing [26]. Furthermore, having more ANC visits might provide mothers with ample time to internalise clinic information but also increase client-provider interactions that can be utilised to remind mothers on the importance of HIV re-testing, which could increase the likelihood of making such a decision. Similar findings have been observed in Burkina Faso and Ethiopia [10, 19].

Good quality of HIV counselling and testing services for expectant mothers was associated with return for re-testing decisions. Quality services build the clients’ trust and make them feel comfortable with the services provided in addition to changing attitude towards the testing, hence the increased likelihood for mothers to go for HIV testing and re-testing [14, 30]. On the other hand, poor counselling might provide inadequate information and, consequently, is less likely to influence the clients to opt for even the first test. Fanta and Worku [27] found that those with an impression of a fair pre-testing counselling were six times more likely to refuse an HIV test than those who had a very good impression. Moreover, those who were unsure about privacy maintenance were more likely to refuse HIV test than those who responded otherwise [27, 31, 32]. Furthermore, positive attitude towards voluntary counselling and testing service had been reported to raise chances of acceptance to test among antenatal care clients in Ethiopia [11] and Congo [30]. Thus, this study highlights the need of our health facilities to improve and maintain quality counselling services coupled with good interaction with clients, confidentiality and privacy to facilitate the uptake of re-testing.

The HIV positivity rate found in this study is high compared to studies done in elsewhere in Africa and within Tanzania [18, 20, 21, 24, 36, 37]. The difference could be explained due to variation of sample size and prevalence of HIV between countries. This present study was conducted among women in the region with high HIV prevalence. Furthermore, the mothers could be in the incubation period during the first test that might result to the negative results hence making them diagnosed on the second test. These results call towards aggressive strategies to reduce risk of transmission to unborn babies from seroconverted mothers [39].

One of the main limitation of this study is that it was done in hospital settings hence not representative of the entire population of pregnant women in the region. The recall bias introduced by the respondents when asking questions related to the services received during their first HIV test could influence the validity of the responses provided, however, most of the information were verified using ANC cards which reduced this bias. Our study design did not allow confirming the tests done hence had to rely on the verbal response and records in the ANC cards. Despite these, we believe that the results are useful to the PMTCT-related services including improving re-testing rate and other aspects related to reproductive health.

## Conclusion

The uptake of HIV re-testing was found to be low among postnatal mothers compared to the Tanzania national target of 90%. This implies looking closely to strengthening existing strategies in preventing mother-to-child HIV transmission particularly among women experiencing seroconversion during pregnancy. The mother’s education level, employment status, number of ANC visits made, reported sexually-transmitted infection, condom use and perceived good quality of the initial HIV test were found to influence the uptake of HIV re-testing among pregnant mothers in the study area. As such, there is a need to strengthen health education and sensitise mothers on the value of re-testing during the ANC visits, outreach services and at delivery room. In addition, the quality of HIV testing and counselling service especially during the first ANC visit should be improved to create conditions that would increase return rates. On the other hand, more epidemiological studies need to be conducted to assess facility system factors that influence the up-take of HIV testing.

## Abbreviations

AIDS: Acquired Immune Deficiency Syndrome
ANC: Antenatal care
AOR: Adjusted Odds Ratio
ART: Antiretroviral Treatment
ARV: Antiretroviral
CCHP: Comprehensive Council Health Plan
CDC: Centre for Disease Control and Prevention
HCW: Healthcare worker
HIV: Human Immunodeficiency Virus
IRB: Institutional Review Board
MLICs: Middle and Low Income Countries
MNCH: Maternal, Neonatal and Child Health
MoHCDGEC: Ministry of Health, Community Development, Gender, Elderly and Children
MUHAS: Muhimbili University of Health and Allied Sciences
PC: Principal Component
PCA: Principal Component Analysis
PEPFAR: President’s Emergency Plan for AIDS Relief
PI: Principal Investigator
PMTCT: Prevention of Mother-to-Child-Transmission
PPS: Probability Proportional to Size
RCHS: Reproductive and Child Health Section
SSA: Sub-Saharan Africa
STI: Sexually-Transmitted Infection
TFELTP: Tanzania Field Epidemiology and Laboratory Training Program
UNAIDS: United Nations Program on AIDS
VCT: Voluntary Counselling and Testing
WHO: World Health Organisation
